# Association of baseline osteocalcin and femoral neck bone mineral density in healthy women with future risk of fractures, cardiovascular disease, diabetes and death

**DOI:** 10.3389/fendo.2025.1652769

**Published:** 2025-11-21

**Authors:** Zheng-Can Pan, Yu-Ying Yang, Xiao-Jing Chen, Tao Jiang, Jia-Xi Song, Chun-Xiang Sheng, Yi-Zhu Wang, Min Xu, Yan-Hua Deng, Guang-Ping Yu, Li-Hao Sun, Hong-Yan Zhao, Jian-Min Liu, Bei Tao

**Affiliations:** 1 Department of Endocrine and Metabolic Diseases, Shanghai Institute of Endocrine and Metabolic Diseases, Ruijin Hospital, Shanghai Jiao Tong University School of Medicine, Shanghai, China; 2 Key Laboratory for Endocrine and Metabolic Diseases of the National Health Commission of the PR China, Ruijin Hospital, Shanghai Jiao Tong University School of Medicine, Shanghai, China; 3 Shanghai Key Laboratory for Endocrine Tumor, Ruijin Hospital, Shanghai Jiao Tong University School of Medicine, Shanghai, China; 4 State Key Laboratory of Medical Genomics, Ruijin Hospital, Shanghai Jiao Tong University School of Medicine, Shanghai, China

**Keywords:** bone metabolism, diabetes, fractures, cardiovascular disease, mortality

## Abstract

**Purpose:**

This study aimed to explore relationships between baseline bone metabolism markers and long-term outcomes, collectively referred to as fractures, cardiovascular diseases, diabetes and all-cause death (FCDD), in healthy women at baseline and identify predictive markers for outcome.

**Methods:**

This study included 356 healthy women and assessed baseline bone turnover markers—osteocalcin, C-telopeptide of type I collagen—as well as bone mineral densities (BMDs) at lumbar spine (L1-4), femoral neck (FN) and total hip (TH). A 16-year retrospective follow-up via telephone questionnaire tracked FCDD occurrence. Statistical tests, including univariate analysis, forward stepwise regression and logistic regression analysis, were used to determine correlations between baseline markers and FCDD.

**Results:**

Among 356 participants, 291 (81.7%) completed follow-up; among these 291 subjects, 109 (37.5%) experienced FCDD. 47 participants experienced fractures (16.2%), 27 developed diabetes (9.3%), 25 experienced cardiovascular events or mortality (8.6%). Stepwise regression identified osteocalcin (odds ratio: 0.938, 95% confidence interval: 0.895–0.980, *P* = 0.006) and FN BMD (odds ratio: 0.066, 95% confidence interval: 0.008–0.490, *P* = 0.009) as independent predictors. However, logistic regression revealed that the protective effect of FN BMD was attenuated after adjusting for age, body mass index, years since menopause, whereas osteocalcin remained significant (*P* < 0.05). Heatmap visualization revealed the lowest FCDD risk among both markers in the highest tertiles (*P* = 0.002).

**Conclusion:**

Our study shows that baseline osteocalcin is independently associated with long-term FCDD outcomes in healthy women. These insights offer valuable guidance for the development of personalized health prevention and intervention strategies.

## Highlights

In a 16-year cohort study of 291 healthy women, baseline osteocalcin and femoral neck (FN) bone mineral density (BMD) were identified as independent predictors of the composite endpoint FCDD (fractures, cardiovascular diseases, diabetes, and death).

Adjustment for confounders attenuated FN BMD’s association, but osteocalcin remained significant; high tertiles of both markers were linked to the lowest FCDD risk (*P* = 0.002), underscoring their predictive value for personalized prevention.

## Introduction

The skeleton is a vital organ in the human body that performs classical functions, such as providing structural support, protecting internal organs, enabling movement, hematopoiesis, mineral storage and metabolism, and fat storage (1). The role of bone metabolism in fractures is well established. Beyond these classical functions, bone also acts as a significant endocrine organ, regulating energy metabolism (2, 3), male reproduction (4), and brain function (5).

In recent years, the relationship between bone and energy metabolism has attracted increasing attention. Osteoblasts modulate systemic energy metabolism by releasing osteocalcin (OCN) and lipocalin-2 (3, 6). Under normal dietary conditions, osteocalcin-knockout mice exhibit reduced insulin secretion, impaired glucose tolerance, and decreased insulin sensitivity (7). In addition, OCN can also regulate fat mass in mice. In osteocalcin-knockout mice, fat mass is increased, the number of adipocytes is higher, and serum triglyceride levels are elevated. Conversely, injecting OCN into wild-type mice leads to a decrease in the above mentioned phenotypes (7–9). Previous studies have reported that the bone formation marker OCN is associated with glucose metabolism parameters and atherosclerotic indices in patients with type 2 diabetes (10). Our previous study showed individuals with higher baseline levels of bone turnover marker C-terminal Telopeptide of Type I Collagen (CTX) in the upper tertile exhibit an increased risk of diabetes and prediabetes over the following four years (11). Similar associations have been reported for other osteogenic markers, further indicating a link between bone and glucose homeostasis (12).

The skeleton also interacts with the cardiovascular system (13). In a study of 986 women (mean age 65 years) with intermediate to high cardiovascular risk (referred to coronary angiography), higher CTX was linked to increased all-cause and cardiovascular mortality, while OCN showed a reverse J-shaped association with noncardiovascular mortality (14). Moreover, OCN has been reported to mediate the crosstalk between bone and cardiovascular health (15). In diabetic rats, supplementation with OCN significantly improves arterial stiffness, angiogenesis, and capillary density and alleviates myocardial fibrosis (16), it also alleviates nonalcoholic fatty liver disease and correlates with kidney function (17, 18). The roles of OCN in regulating muscle strength, cognition, and behavior abnormalities in Alzheimer’s disease (AD) and Parkinson’s disease (PD) mice and rat models have also been documented (19–21).

Fractures, CVD, and diabetes often co-occur in elderly individuals and are associated with increased mortality (22–25). Given the involvement of bone in these disorders, we aimed to investigate whether bone-related biomarkers and bone mineral densities (BMDs) are predictive of these events. In this study, we defined a composite endpoint, FCDD (fractures, CVD, diabetes, and all-cause death), to represent this cluster of diseases. The combination of FCDD is based on the consideration that these four endpoints, although presenting with distinct phenotypes, have common risks and share core pathophysiological foundations, such as aging, menopause (26, 27), chronic low-grade inflammation (28, 29), and energy metabolism dysregulation (30), supporting FCDD as a reasonable composite endpoint. However, existing studies only focus on single and/or dual outcomes, lacking unified predictive indicators for the composite endpoint. Thus, we aimed to examine the associations between baseline bone metabolism biomarkers, along with BMDs and the occurrence of FCDD in a cohort of healthy women, with a median age of 56 years and 16 years of follow-up.

## Methods

### Study design and participants

In 2007, We established a study cohort of 1,012 women (31). Baseline information for all participants was systematically collected through face-to-face interviews using a structured questionnaire. The questionnaire focused on key aspects, such as socioeconomic status (including age, years since menopause [YSM], and medical history), lifestyle factors, including smoking, alcohol consumption, and coffee consumption; and health-related variables, such as calcium or vitamin D supplementation and history of hypertension. Body weight and height were measured, and body mass index (BMI) was calculated.

Blood biochemical markers, including serum levels of OCN, CTX, calcium, and phosphorus, were measured, and the BMDs at lumbar-spine 1-4 (L1-4), femoral neck (FN) and total hip (TH) were measured using dual-energy X-ray absorptiometry (DXA) (31, 32). During the initial survey, the participants were also told about the planned long-term follow-up, but only 356 of them consented to continued surveillance. Subsequent analyses were conducted in this subset of subjects who agreed to follow-up.

To evaluate the representativeness of the 356 participants who consented to follow-up (vs. the initial 1,012 women), we conducted a comparative analysis of baseline characteristics between the two groups. Detailed comparison results are presented in **Supplementary Table 1**.

### Data collection and definition

During this follow-up study, the participants (or the relatives of those who had passed away) were asked through phones whether they had experienced FCDD in the past 16 years. For the diagnosis of diabetes, both the type and time of diagnosis were recorded. Fractures were defined as those occurring when the trauma is equivalent to that generated by a fall from a standing height or lower, especially in the past year. CVD encompasses a heart attack and/or myocardial infarction. All-cause mortality encompassed all deaths during the follow-up period, regardless of the underlying causes. To verify the credibility of self-reported FCDD outcomes, we simultaneously collected participants’ medication history during telephone follow-up. Specifically, participants who self-reported diabetes were asked to report the use of anti-diabetic drugs (e.g., metformin, insulin); those who self-reported CVD were asked to report the use of lipid-lowering or anti-hypertensive drugs (e.g., statins, angiotensin-converting-enzyme-inhibitors); and those who self-reported fractures were asked to provide details of fall history. We cross-validated the consistency between self-reported outcomes and medication/fall history to reduce recall bias.

### Statistical analysis

Statistical analyses were performed via IBM SPSS Statistics 25 (version 9.2) and R (version 4.0.5). Continuous variables were reported as means ± SDs for normally distributed data or medians (interquartile ranges, IQRs) for skewed distributions. Comparisons between two groups were performed using independent-samples t tests and Mann–Whitney U tests, as appropriate. Categorical variables were presented as percentages, and difference between two groups were assessed using chi-square (χ2) tests.

Univariate logistic regression analysis was used to assess the associations between baseline variables and FCDD and its components, with results expressed as odds ratios (ORs) and 95% confidence intervals (CIs). To identify factors independently associated with FCDD, multivariate regression analysis was performed. This model included all variables that showed a significant association with FCDD or its components in the univariate analysis, together with all bone-related parameters of interest—such as BMD and serum bone turnover markers—regardless of their significance in the univariate analysis. A P-value of less than 0.05 was considered statistically significant.

## Results

### Clinical features of the participants involved in this study

**Figure 1:**
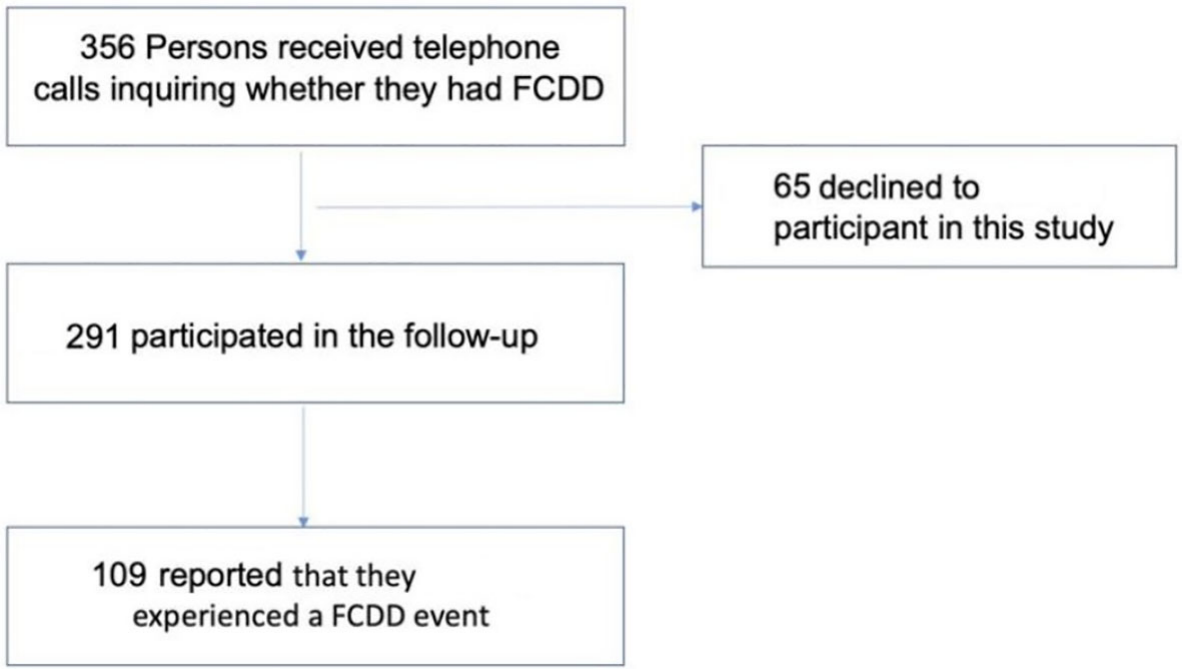
The selection process for the study cohort.

In 2023, a total of 356 individuals were contacted by phone to inquire whether they had experienced any FCDD events during the past 16 years following their initial enrollment in 2007. Of these, 65 (18.3%) declined to participate in the follow-up survey. Consequently, 291 individuals (81.7%) were ultimately included in this study (**Figure 1**). Comparison of baseline characteristics between the original 356 individuals and the 291 follow-up individuals revealed no significant differences across any parameters (*P* > 0.05), including age, BMI, YSM and other key factors (**Supplementary Table 2**).

### FCDD events and baseline characteristics

Of the 291 participants, 109 (37.5%) experienced FCDD events over the 16-year follow-up period. Baseline characteristics of the FCDD and non-FCDD groups were presented in **Table 1**. Notably, there was a significant reduction in the BMD at L1–4 in FCDD group (0.993 g/cm^2^ vs. 1.050g/cm^2^, *P* = 0.03). Compared with non-FCDD group, a trend toward lower serum OCN levels was observed in the FCDD group (17 ng/ml vs. 18 ng/ml, *P* = 0.05), FN BMD also tended to be lower(0.823 g/cm^2^ vs. 0.850 g/cm^2^, *P* = 0.07). For other variables, such as age (*P* = 0.14), BMI (*P* = 0.39), YSM (*P* = 0.10), and other measured indices (fall, hypertension, etc.), no significant differences were found between the two groups (all *P >*0.05).

**Table 1:** Comparison of baseline characteristics between non-FCDD participants and FCDD participants.

	non-FCDD(n=182)	FCDD(n=109)	
Variables	Median (Q1, Q3)/Percentage (%)	Median (Q1, Q3)/Percentage (%)	p_value
Age(years)	55(52, 60)	56(53, 62)	0.14
BMI(kg/m^2^)^a^	22.77(21.12, 24.76)	23.15(20.96, 25.61)	0.39
YSM(years)^b^	5(1, 10)	6(3, 12)	0.10
Fall(%)	30.5	31.5	0.89
Hypertension(%)	14.29	15.60	0.86
Smoking(%)	0.55	0.92	1
Alcohol Drinking(%)	2.75	0.92	0.42
Tea drinking(%)	24.18	22.02	0.77
Coffee drinking(%)	15.38	16.51	0.87
Calcium supplementation(%)	21.43	22.94	0.77
VitD supplementation(%)	3.85	3.67	1
Serum calcium(mmol/L)	2.32(2.20, 2.39)	2.31(2.20, 2.41)	0.93
Serum phosphorus(mmol/L)	1.24(1.14, 1.38)	1.22(1.09, 1.38)	0.54
Serum osteocalcin(ng/mL)	18(15, 22)	17(14, 21)	0.05
Serum CTX(ng/mL)^c^	0.40(0.29, 0.53)	0.38(0.30, 0.50)	0.28
BMDs(g/cm^2^)			
L1-4^d^	1.050(0.914, 1.168)	0.993(0.881, 1.090)	0.03*
FN^e^	0.850(0.772, 0.944)	0.823(0.737, 0.908)	0.07
TH^f^	0.923(0.827, 1.008)	0.870(0.790, 1.010)	0.14

^a^Body mass index; ^b^years since menopause; ^c^C-terminal telopeptide of type I collagen; ^d^Lumbar spine 1-4; ^e^femoral neck; ^f^total hip.

*p<0.05.

### Factors responsible for the occurrence of FCDD

Univariate regression analyses were performed for the composite FCDD and its component using all baseline parameters. For FCDD, variables such as YSM (*P* = 0.07) and serum OCN (*P* = 0.06) showed trends close to significance. For FCDD individual component, age, BMI, OCN, BMDs were significantly related with diabetes, fractures and CVD/Death (**Supplementary Table 3**).

Multivariate stepwise regression analysis identified osteocalcin (OCN) [OR 0.938, 95% CI 0.895 to 0.980; P = 0.006] and femoral neck BMD (FN BMD) [OR 0.066, 95% CI 0.008 to 0.490; P = 0.009] as significant factors associated with FCDD. The model explained 4.2% (Cox-Snell R²) to 5.9% (Nagelkerke R²) of the variance.

Since several factors, such as age, BMI and YSM are known to influence fracture, diabetes and CVD, their potential confounding effects should be examined. As demonstrated in **Table 2**, in unadjusted Model 1, both OCN [OR 0.952 (0.911, 0.994), *P* = 0.027] and FN BMD [OR 0.126 (0.018, 0.880), *P* = 0.037] were significantly associated with FCDD. In Model 2, after adjusting for age and BMI, the significance of FN BMD disappeared (*P* = 0.104), whereas OCN still maintained a significant association [OR 0.946 (0.905, 0.990), *P* = 0.017]. In Model 3, after adjusting for YSM, FN BMD remained nonsignificant (*P* = 0.164), whereas OCN continued to be a significant predictor [OR 0.947, (0.905, 0.991), *P* = 0.018].

**Table 2:** Logistic regression analysis of the associations between osteocalcin, FN BMD and FCDD.

		OR(95%CI)	p_value
Model1	Osteocalcin	0.952(0.911, 0.994)	0.027
	FN BMD	0.126(0.018, 0.880)	0.037
Model2	Osteocalcin	0.946(0.905, 0.990)	0.017
	FN BMD	0.145(0.014, 1.486)	0.104
Model3	Osteocalcin	0.947(0.905, 0.991)	0.018
	FN BMD	0.184(0.017, 1.998)	0.164

Model 1: unadjusted.

Model 2: adjusted for age and BMI.

Model 3: adjusted for age, BMI and YSM.

To further explore the individual and combined associations of these two markers on FCDD, participants were then stratified into three groups based on the tertiles of OCN and FN BMD, respectively, and into nine groups representing all possible combinations of these tertiles.

**Figure 2:**
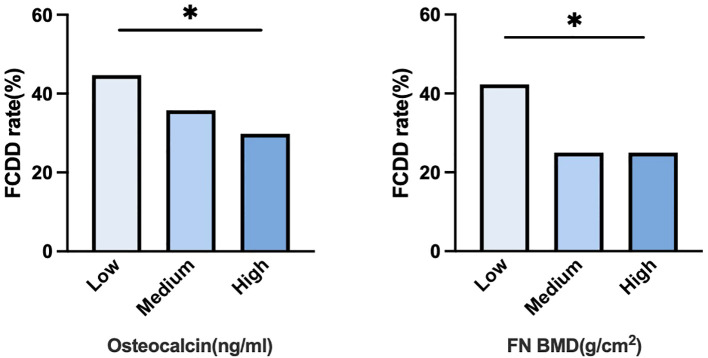
FCDD rates across different levels of osteocalcin and FN BMD. * p<0.05.

**Figure 3:**
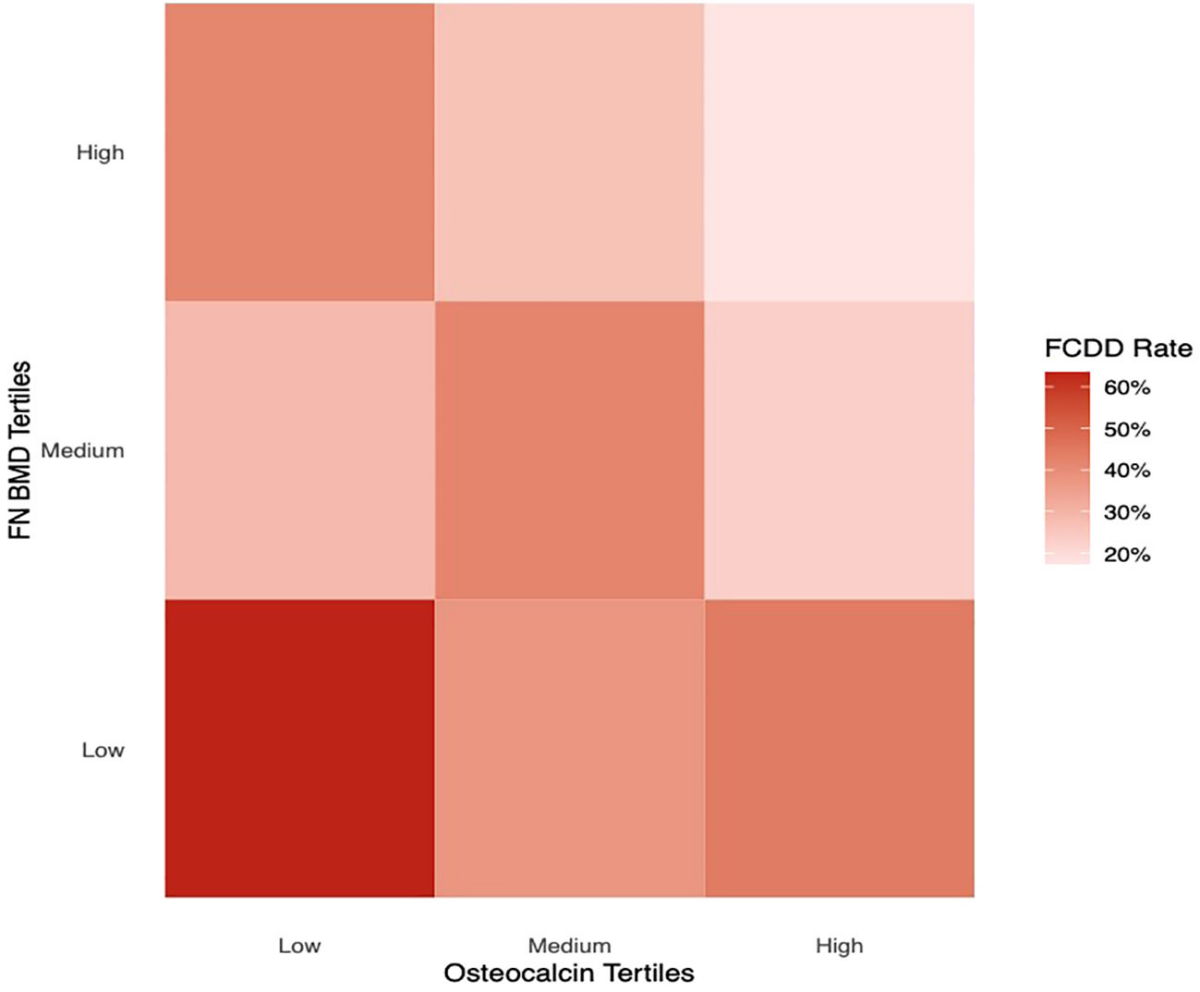
FCDD rates across combinations of osteocalcin and FN BMD thresholds.

**Figure 2** illustrated the FCDD incidence rates across OCN and FN BMD tertiles (low, medium, and high). The FCDD rate was significantly higher in the low OCN tertile than in the high tertile. Similarly, participants in the low FN BMD tertile exhibited a significantly higher FCDD rate compared with those in high tertile. The heatmap provided a visual exploration of the combined patterns of OCN and FN BMD. As shown in **Figure 3**, the lowest FCDD rate was observed when both OCN and FN BMD were in their highest tertiles. Conversely, the FCDD rate was the highest when both were in the lowest tertiles. Fisher’s exact test confirmed a statistically significant difference between these two extreme groups (*P* = 0.002).

## Discussion

In this 16-year longitudinal cohort of 291 healthy women, OCN emerged as a independent predictor of the composite endpoint FCDD (fractures, cardiovascular diseases, diabetes and death) over 16 years. After adjustment for age, BMI, and YSM, each unit increase in serum OCN level was associated with a 5.3% reduction in FCDD risk. Compared with participants in the lowest tertile, those in the highest tertiles of both serum OCN and the FN BMD at baseline exhibited the lowest FCDD risk.

The association between OCN and FCDD events observed in our study aligns with its multifaceted physiological roles in modulating glucose metabolism (33), muscle strength (34), cardiovascular health, including acute cardiovascular events such as myocardial infarction and all-cause mortality (35, 36). The direct effects of OCN on energy metabolism by promoting insulin secretion, improving insulin resistance, and predicting diabetes and its complications have been widely reported in basic research and both cross-sectional and longitudinal clinical studies, and have been comprehensively reviewed (37–39). Emerging evidence also supports a role for OCN in cardiovascular regulation. Mechanistically, OCN modulates autonomic nervous system activity by enhancing sympathetic tone and inhibiting parasympathetic tone, thereby facilitating cardiovascular adaptation (36). OCN also plays a critical role in stress responses. As shown by Berger et al., it is rapidly secreted during acute stress to regulate parasympathetic activity, thereby facilitating essential physiological changes, including increased heart rate, respiratory efficiency, and energy expenditure (40). These findings suggest that OCN can mediate adaptive stress responses, which may contribute to the integration of bone metabolism with broader systemic homeostasis. Overall, the association between the baseline serum OCN level and FCDD events during follow-up found in this study is biologically plausible, supported by existing evidence of OCN’s roles in glucose metabolism and cardiovascular homeostasis —though our observational design cannot confirm causality (12, 41).

With respect to FN BMD, its association with FCDD weakened after adjusting for covariates including age, BMI, and YSM, indicating that its effect is confounded by age-, estrogen- and/or obesity-related factors. Indeed, the pronounced influences of age, BMI and YSM on the development of fracture, diabetes and CVD are anticipated and well-established. For instance, as shown in a population-based epidemiological study with a sample size of 17,208 people, with increasing age, the prevalence of osteoporosis defined by FN BMD increased from 5.3% in women aged 50–59 years to 16.8% in those aged 60–69 years (42), suggesting the powerful effect of age on osteoporosis. Age also affects vascular properties, contributing to increased stiffness, an early marker of CVD (43, 44).

With respect to BMI, growing evidence supports its role in mediating diabetes, fracture and CVD risk (45). Clinical studies revealed that each unit increase in BMI is associated with a 109% higher risk of developing T2DM (45), while weight reduction has consistently been linked to lower risks of both diabetes and CVD (46). Most importantly, obesity-related chronic low-grade inflammation is mechanically responsible for osteoporosis, diabetes and CVD (28, 29). Obese individuals exhibit increased activation of Toll-like receptor 2 (TLR2) on monocytes, driving the polarization of anti-inflammatory M2 macrophages to the proinflammatory M1 phenotype. This transition leads to the upregulation of cytokines such as IL-6 and TNF-α, which stimulate osteoclast differentiation and promote bone matrix degradation (47), thereby potentially increasing fracture risk. Furthermore, a mouse study showed that increased bone resorption activity is responsible for arterial stiffness (48). Concurrently, inflammation can induce endothelial dysfunction and lipid deposition, directly contributing to cardiovascular disease. Collectively, these findings are helpful to explain why, after adjusting for BMI, the association between FN BMD and FCDD is attenuated. They also indicate that, in terms of reducing the risk of diabetes and CVD, BMI plays a more important role than does BMD, although a higher BMI is positively correlated with BMD due to mechanical loading effects.

In our analysis, the protective effect of FN BMD on FCDD weakened after adjusting for YSM, indicating that estrogen deficiency and/or YSM play important roles in FCDD. Importantly, estrogen is well recognized for its cytoprotective effects. Estrogen acts on cardiomyocytes and endothelial cells by inhibiting apoptosis and necrosis and modulating these critical shared pathways. Clinically, postmenopausal estrogen deficiency results in BMD loss, which is closely associated with YSM (49), and the indirect association of BMD with cardiovascular outcomes through estrogen-mediated pathways has also been described (50). In addition, loss of hip BMD is significantly associated with higher coronary heart disease mortality in women aged 65 years and older (relative hazard [RH] = 1.3) (51). It was shown that among elderly postmenopausal women with a longer YSM, the impact of decreased bone mineral content at menopause on cardiovascular mortality is weaker than that among younger women with a shorter duration of menopause (52). In addition, higher risks of fatal and nonfatal CVD were also found in women with 5 to 10, 10 to 15, 15 to 20, or >20 years since menopause compared with <5 years since menopause (53). These findings may explain why BMD no longer serves as an independent predictor of CVD in female cohorts, with YSM emerging as the primary predictive factor. Together, these findings highlight the complex interplay among BMD, age-related biological decline, and obesity-related metabolic inflammation in determining the risk of FCDD composite endpoint.

An important finding of this study, illustrated by our heatmap analysis, is that participants with baseline serum OCN and FN BMD in the highest tertiles exhibited the lowest FCDD risk during follow-up. It is not clear why there is such a combination. Notably, OCN serves as a marker of bone formation and remodeling, while also exerting diverse endocrine effects that contribute to overall human health. BMD is also an objective parameter of bone health. Our findings support the hypothesis that the combined assessment of bone turnover activity (OCN) and bone structural integrity (BMD) may provide a more comprehensive risk prediction framework for FCDD than either parameter alone.

Notably, the construction of FCDD as a composite endpoint is scientifically justified: first, its components (fractures, CVD, diabetes, all-cause death) share age-related epidemiological trends (prevalence/incidence rising with age, frequent multimorbidity in the elderly); second, they share core pathophysiological mechanisms (e.g., obesity-related chronic low-grade inflammation, energy metabolism disorders involving OCN, postmenopausal estrogen deficiency); third, it has clinical value for guiding multimorbidity prevention (e.g., cross-disease effects of anti-osteoporotic or anti-diabetic drugs) (54, 55). FCDD facilitates integrated management of age-related conditions: anti-osteoporotic drugs may improve vascular function (53) and are beneficail for glucose metabolism (56), while anti-diabetes drug metformin increases BMD (54) and improve bone quality (57), reflecting shared therapeutic targets. Moreover, FCDD enables integrated risk assessment—markers like OCN predicting FCDD can act as simple biomarkers to identify high-risk individuals, addressing unmet multimorbidity stratification needs.

We acknowledge FCDD’s inherent heterogeneity: fractures, CVD, and diabetes involve distinct pathways, with the same marker exerting divergent effects. For example, age drives linear fracture risk in the general population but not in T2DM (reshaped by diabetes-specific factors) (58); high HDL-C protects against CVD yet may increase fracture risk (59); hyperuricemia raises CVD risk but lowers fracture risk via antioxidant effects (60, 61). Our study focused on FCDD’s overall association to capture multimorbidity burden, while future research could dissect marker associations with individual components to clarify specific mechanisms.

Despite these contributions, limitations of this study include the following: 1) Retrospective design with potential residual confounding; 2) Relatively small sample size and selection bias—initially, 1,012 women were enrolled in the cohort, while only 356 consented to long-term follow-up. To address this concern, we compared baseline characteristics between the 356 consenting follow-up participants and the initial 1,012-cohort. Results showed no statistically significant differences in all key variables (all *P >*0.05), as detailed in **Supplementary Table 1**, suggesting minimal impact of selection bias and the representativeness of the follow-up group; 3) The endpoints of FCDD were all self-reported but not confirmed with medical documents. However, we mitigated these biases by collecting auxiliary information (e.g., medication use, fall history) during telephone follow-up for cross-validation with self-reported outcomes. Additional limitations include lack of baseline lipid data.

This study is the first study linking FCDD with OCN/FN BMD. We suggest monitoring, with future studies supplementing lipid data to clarify mechanisms.

In conclusion, the present study provides new evidence suggesting an association between bone metabolism and the development of FCDD. Monitoring bone metabolism in perimenopausal women may help guide the management of multiple age-related diseases. Considering that bone health can reflect overall systemic health, future large-scale prospective studies are needed to validate our findings, elucidate the molecular mechanisms underlying OCN/FN BMD–FCDD relationships, and perform interventional trials targeting OCN/BMD pathways to reduce FCDD risk.

## Data Availability

The original contributions presented in the study are included in the article/**Supplementary Material**. Further inquiries can be directed to the corresponding authors.
