# Supplementary material for: Association of baseline osteocalcin and femoral neck bone mineral density in healthy women with future risk of fractures, cardiovascular disease, diabetes and death
Source: Front Endocrinol (Lausanne). 2025 Nov 21;16:1652769. doi: 10.3389/fendo.2025.1652769 (PMC12678083; doi:10.3389/fendo.2025.1652769)
Supplement: Supplementary file 1 [file Table1.docx]

**Supplementary Table 1**. The characteristics between initial total cohort and consenting follow-up cohort

| Variables | Consenting follow-up cohort (n=356) | Initial total cohort(n=1012) | p_value |
| --- | --- | --- | --- |
| Age(years) | 56 (53, 62) | 57 (53, 63) | 0.0926 |
| BMI (kg/m^2^) ^a^ | 22.89 (20.96, 23.21) | 22.89 (20.96, 25.11) | 0.6457 |
| YSM (years)^b^ | 5(1,11) | 6(2,13) | 0.0717 |
| Fall (%) | 32.3 | 33.0 | 0.8599 |
| Hypertension (%) | 15.2 | 18.5 | 0.1815 |
| Smoking (%) | 0.8 | 0.9 | 1 |
| Alcohol drinking (%) | 2.5 | 1.8 | 0.5154 |
| Tea drinking (%) | 25.3 | 21.1 | 0.1258 |
| Coffee drinking (%) | 16.3 | 12.6 | 0.0931 |
| Calcium supplementation  (%) | 21.1 | 22.4 | 0.6676 |
| VitD supplementation (%) | 3.1 | 2.2 | 0.4425 |
| Serum calcium(mmol/l) | 2.32 (2.20, 2.41) | 2.32 (2.20, 2.41) | 0.4415 |
| Serum phosphorus(mmol/l) | 1.24 (1.12, 1.38) | 1.27 (1.14, 1.39) | 0.1328 |
| Serum osteocalcin (ng/ml) | 18.00 (15.00, 22.00) | 18.00 (15.00, 23.00) | 0.0735 |
| Serum CTX-1 (ng/ml) ^c^ | 0.41 (0.30, 0.53) | 0.42 (0.30, 0.56) | 0.2442 |
| BMDs(g/cm^2^) | | | |
| L1-4^d^ | 1.03 (0.91, 1.15) | 1.01 (0.90, 1.13) | 0.1718 |
| FN^e^ | 0.84 (0.75, 0.94) | 0.84 (0.74, 0.92) | 0.1290 |
| TH^f^ | 0.91 (0.81, 1.01) | 0.90 (0.80, 0.99) | 0.0994 |

a. body mass index; b. years since menopause; c. C-terminal Telopeptide of Type I Collagen;

d. lumbar spine 1-4; e. femur neck; f. total hip
