# Supplementary material for: Association of baseline osteocalcin and femoral neck bone mineral density in healthy women with future risk of fractures, cardiovascular disease, diabetes and death
Source: Front Endocrinol (Lausanne). 2025 Nov 21;16:1652769. doi: 10.3389/fendo.2025.1652769 (PMC12678083; doi:10.3389/fendo.2025.1652769)
Supplement: Supplementary file 2 [file Table2.docx]

**Supplementary Table 2.** The characteristics between follow-up cohort and total cohort.

| Variables | Total cohort(n=356) | Follow-up cohort(n=291) | p_value |
| --- | --- | --- | --- |
| Age(years) | 56 (53, 62) | 55 (53, 61) | 0.7037 |
| BMI (kg/m^2^)^a^ | 22.89 (20.96, 23.21) | 22.83 (20.96, 23.24) | 0.8230 |
| YSM (years)^b^ | 5(1,11) | 5(1,11) | 0.8489 |
| Fall (%) | 32.3 | 31.3 | 0.8450 |
| Hypertension (%) | 15.2 | 14.8 | 0.9775 |
| Smoking (%) | 0.8 | 0.7 | 1 |
| Alcohol drinking (%) | 2.5 | 2.1 | 0.8970 |
| Tea drinking (%) | 25.3 | 23.4 | 0.6373 |
| Coffee drinking (%) | 16.3 | 15.8 | 0.9527 |
| Calcium supplementation  (%) | 21.1 | 22.0 | 0.8501 |
| VitD supplementation (%) | 3.1 | 3.8 | 0.7919 |
| Serum calcium(mmol/l) | 2.32 (2.20, 2.41) | 2.31 (2.20, 2.40) | 0.8476 |
| Serum phosphorus(mmol/l) | 1.24 (1.12, 1.38) | 1.23 (1.13, 1.38) | 0.6520 |
| Serum osteocalcin (ng/ml) | 18.00 (15.00, 22.00) | 18.00 (15.00, 22.00) | 0.8086 |
| Serum CTX-1 (ng/ml)^c^ | 0.41 (0.30, 0.53) | 0.40 (0.30, 0.52) | 0.6593 |
| BMDs (g/cm^2^) | | | |
| L1-4^d^ | 1.03 (0.91, 1.15) | 1.03 (0.90, 1.15) | 0.9356 |
| FN^e^ | 0.84 (0.75, 0.94) | 0.84 (0.76, 0.93) | 0.7647 |
| TH^f^ | 0.91 (0.81, 1.01) | 0.91 (0.81, 1.01) | 0.8032 |

a. body mass index; b. years since menopause; c. C-terminal Telopeptide of Type I Collagen;

d. lumbar spine 1-4; e. femur neck; f. total hip
