# Supplementary material for: Association of baseline osteocalcin and femoral neck bone mineral density in healthy women with future risk of fractures, cardiovascular disease, diabetes and death
Source: Front Endocrinol (Lausanne). 2025 Nov 21;16:1652769. doi: 10.3389/fendo.2025.1652769 (PMC12678083; doi:10.3389/fendo.2025.1652769)
Supplement: Supplementary file 3 [file Table3.docx]

**Supplementary Table 3.** Univariate Regression Analysis of Single and Composite Outcomes (FCDD, Diabetes, Fractures, and CVD/Death).

|  | **FCDD** | **Diabetes** | **Fracture** | **CVD/Death** |
| --- | --- | --- | --- | --- |
| **Variables** | **OR (95% CI)** | **OR (95% CI)** | **OR (95% CI)** | **OR (95% CI)** |
| Age (years) | 1.023 (0.995, 1.052) | 1.028 (0.979, 1.081) | 0.985 (0.95, 1.023) | **1.154 (1.095, 1.224)** *** |
| BMI (kg/m^2^)^a^ | 1.039 (0.961, 1.125) | **1.305 (1.14, 1.506)** *** | **0.866 (0.77, 0.968)** * | 1.081 (0.946, 1.233) |
| YSM (years)^b^ | 1.029 (0.998, 1.062) | 1.012 (0.954, 1.067) | 0.999 (0.951, 1.046) | **1.133 (1.081, 1.192)** *** |
| Fall (%) | 1.141(0.685, 1.889) | 0.781(0.306, 1.805) | 0.936(0.466, 1.803) | 1.839 (0.797, 4.171) |
| Hypertension (%) | 1.117(0.572, 2.141) | 1.834(0.664, 4.501) | 0.538(0.167, 1.373) | **3.162(1.245,7.565)*** |
| Smoking (%) | 1.673 (0.173, 16.250) | 1.914 (0.093, 39.488) | 1.021 (0.052, 19.673) | 10.807 (0.413, 284.233) |
| Alcohol drinking (%) | 0.446 (0.070, 2.857) | 0.722 (0.039, 13.322) | 1.405 (0.233, 8.743) | 0.785 (0.042, 14.698) |
| Tea drinking (%) | 0.892(0.503, 1.555) | 0.979(0.361, 2.346) | 0.457(0.174, 1.032) | 1.355(0.522, 3.208) |
| Coffee drinking (%) | 1.096(0.570, 2.063) | 1.317(0.444, 3.331) | 0.791(0.297, 1.823) | 1.106(0.335, 2.971) |
| Calcium supplementation (%) | 1.096(0.617, 1.923) | 1.608(0.650, 3.690) | 1.128(0.526, 2.284) | 1.812(0.725, 4.223) |
| VitD supplementation (%) | 0.998(0.297, 3.347) | 0.400 (0.008, 19.365) | 3.266 (0.977, 10.952) | 0.436(0.009, 20.94) |
| Serum calcium (mmol/l) | 1.37 (0.375, 5.081) | 3.251 (0.38, 29.942) | 2.446 (0.41, 15.515) | 7.587 (0.83, 74.431) |
| Serum phosphorus (mmol/l) | 0.549 (0.137, 2.16) | 0.431 (0.047, 3.86) | 1.035 (0.159, 6.765) | 1.068 (0.108, 10.737) |
| Serum osteocalcin (ng/ml) | 0.963 (0.924, 1.001) | **0.9 (0.824, 0.972)** * | 0.991 (0.941, 1.041) | 0.957 (0.883, 1.027) |
| Serum CTX-1 (ng/ml)^c^ | 0.407 (0.1, 1.576) | **0.031 (0.002, 0.431)** * | 0.83 (0.132, 4.673) | 0.401 (0.028, 4.302) |
| **BMDs (g/cm^2^)** |  |  |  |  |
| L1-4^d^ | 0.374 (0.099, 1.368) | **12.169 (1.507, 101.149)** * | 0.228 (0.036, 1.321) | **0.018 (0.001, 0.227)** ** |
| FN^e^ | 0.234 (0.037, 1.429) | 13.039 (0.672, 261.289) | **0.036 (0.003, 0.439)** * | **0.003 (0, 0.088)** *** |
| TH^f^ | 0.327 (0.057, 1.812) | **23.053 (1.319, 431.463)** * | **0.036 (0.003, 0.402)** ** | **0.012 (0, 0.278)** ** |

a. body mass index; b. years since menopause; c. C-terminal Telopeptide of Type I Collagen;

d. lumbar spine 1-4; e. femur neck; f. total hip

^*^p < 0.05；^**^p < 0.01；^***^p < 0.001
